# Editorial: Advancing immune research in chronic liver diseases through new approach methodologies

**DOI:** 10.3389/fimmu.2026.1912152

**Published:** 2026-06-30

**Authors:** Sara Massironi, Ana C. Maretti-Mira

**Affiliations:** 1 Medicine and Surgery, Vita-Salute San Raffaele University, Milan, Italy; 2 Keck School of Medicine, University of Southern California, Los Angeles, CA, United States

**Keywords:** chronic liver diseases (CLD), immunometabolism, liver immunology, single-cell technologies, spatial transcriptomics (ST)

Chronic liver diseases (CLDs), including autoimmune liver diseases, metabolic dysfunction-associated steatotic liver disease (MASLD), and progressive fibrotic disorders, remain a major global health burden (1–4). Despite substantial advances in hepatology, the complex interplay between immune responses, metabolic pathways, and tissue remodeling continues to challenge both mechanistic understanding and clinical management (5, 6). In recent years, the emergence of innovative methodological approaches has provided unprecedented opportunities to dissect disease heterogeneity, identify novel biomarkers, and develop more precise diagnostic and therapeutic strategies (5, 7).

The Research Topic “Advancing Immune Research in Chronic Liver Diseases Through New Approach Methodologies” was conceived to highlight these advances and to showcase how emerging technologies are reshaping our understanding of liver immunopathology (8–10). The contributions collected in this Topic span autoimmune liver diseases, hepatic fibrosis, and MASLD, illustrating the growing convergence of immunology, systems biology, computational sciences, and non-invasive clinical assessment.

A major theme emerging from this Research Topic is the increasing ability to characterize the immune microenvironment at high resolution (10, 11). One contribution (Liu et al.) highlights how single-cell sequencing, spatial transcriptomics, and organoid models are transforming research in chronic liver diseases and hepatocellular carcinoma. These technologies enable the identification of previously unrecognized immune cell subsets, reveal spatially organized cellular interactions, and provide experimental platforms that more faithfully reproduce human disease biology (10–13). The integration of these state-of-art technologies enables a paradigm shift from static descriptions of immune cell populations toward dynamic and spatially resolved analyses of disease mechanisms. More importantly, these approaches challenge the traditional view of chronic liver diseases as homogeneous clinicopathological entities, revealing instead complex cellular ecosystems whose composition, functional states, and spatial organization may ultimately determine disease behavior and therapeutic responsiveness.

The importance of integrating immunological and metabolic information is further highlighted by the work of Xiao et al., who investigated glycolysis-related molecular signatures during hepatic fibrosis progression. Using transcriptomic datasets, machine-learning algorithms, and experimental validation, the authors identified glycolysis-associated clusters characterized by distinct immune infiltration profiles. Their findings reinforce the concept that metabolic reprogramming is not merely a consequence of chronic liver injury but actively shapes immune responses and fibrogenesis. Such integrative approaches may ultimately facilitate the identification of novel therapeutic targets linking metabolism and immunity. Beyond their immediate mechanistic implications, these findings align with a broader shift in liver immunology toward the recognition that metabolic programs actively determine immune-cell phenotypes and tissue responses. In this context, metabolic rewiring emerges not simply as a consequence of chronic inflammation, but as a fundamental regulator of immune-cell fate and fibrogenic activity. This evolving perspective is progressively blurring the traditional boundaries between metabolism and immunity, supporting the concept of immunometabolism as a central driver of liver disease progression and a promising source of future therapeutic targets.

Another key aspect addressed by this Topic is the growing recognition of shared autoimmune mechanisms across organ systems (14). A contribution (Massironi et al.) explored the overlap between autoimmune gastritis and autoimmune liver diseases, revealing a clinically relevant association, particularly among patients with primary biliary cholangitis (15). The high prevalence of autoimmune gastritis and associated gastric premalignant lesions supports the need for broader surveillance strategies and suggests common immunopathogenic pathways that extend beyond individual organs. These observations further support the concept that apparently organ-specific autoimmune diseases frequently arise within broader networks of immune dysregulation that transcend anatomical boundaries, reinforcing the need for a more integrated view of autoimmunity.

The search for accessible and clinically useful biomarkers remains another priority in CLDs research. In this regard, Chen et al. reviewed the available evidence linking serum immunoglobulins with MASLD progression. Although the available evidence remains heterogeneous, the review highlights the emerging role of humoral immune responses in metabolic liver disease. These observations support the concept that immune dysregulation is not merely a consequence of metabolic dysfunction but may actively contribute to disease progression and fibrosis development. Similarly, Huang et al. addressed a critical challenge in autoimmune hepatitis: the interpretation of liver stiffness measurements in the presence of active inflammation. Their study demonstrated that inflammatory activity significantly influences elastography-based fibrosis assessment and proposed a correction model capable of improving diagnostic accuracy. Importantly, these findings challenge the assumption that non-invasive biomarkers directly reflect a single pathological process. Instead, they emphasize the need to interpret diagnostic tools within their biological context, acknowledging the influence of inflammatory activity on fibrosis assessment and disease stratification, and enhance clinical decision-making (16). Together, the studies by Chen and Huang underscore a broader methodological challenge: biomarkers rarely represent isolated biological processes. Instead, they often reflect the integrated consequences of immune activation, metabolic perturbation, and tissue remodeling. Interpreting these signals, therefore, requires a deeper understanding of the biological context in which they arise.

**Figure 1:**
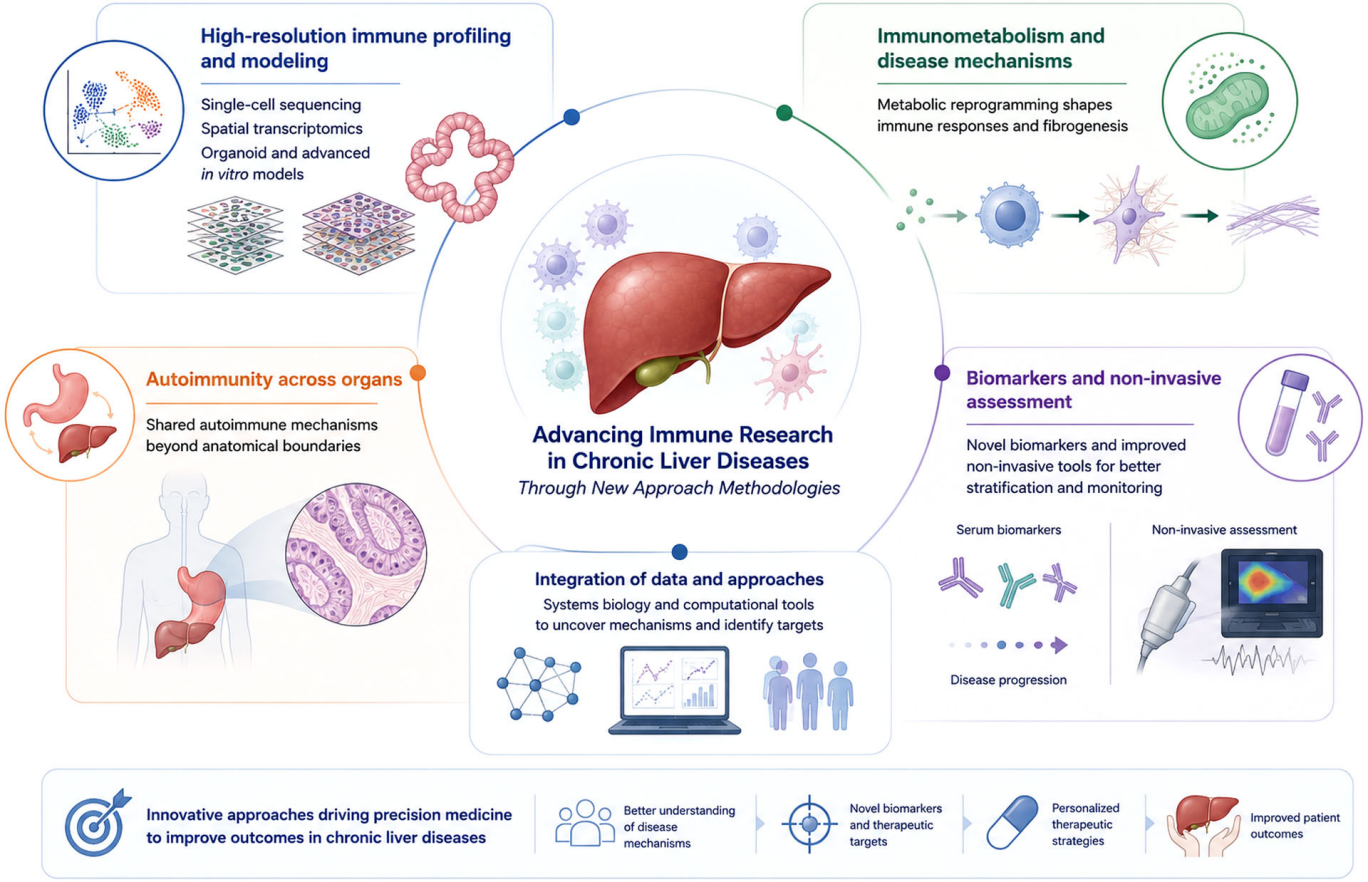
Conceptual overview of emerging methodologies advancing immune research in chronic liver diseases.

Taken together, the studies included in this Research Topic demonstrate how innovative methodologies are driving a deeper understanding of chronic liver diseases (**Figure 1**). Beyond generating descriptive data, these approaches are increasingly enabling mechanistic insights, improved patient stratification, and more accurate disease monitoring. Importantly, they highlight the growing convergence between advanced omics technologies, computational modeling, immunological profiling, and translational clinical research.

Looking forward, the integration of multi-omics datasets, advanced *in vitro* models, artificial intelligence-based analytical tools, spatially resolved technologies, and validated non-invasive biomarkers is poised to accelerate the transition toward precision hepatology (5, 7, 10). Collectively, these advances suggest that the future of hepatology may rely less on conventional disease classifications and increasingly on biologically informed patient stratification based on immune, metabolic, spatial, and molecular signatures. Ultimately, the challenge ahead will not simply be the generation of increasingly large datasets, but their integration into biologically coherent models capable of explaining patient-specific disease trajectories and individualizing diagnostic and therapeutic strategies (9, 17). The studies collected in this Research Topic exemplify how methodological innovation is progressively transforming chronic liver diseases from broadly defined clinical entities into biologically stratified conditions, thereby laying the foundation for precision hepatology.
